# ATF4/TXNIP/REDD1/mTOR signaling mediates the antitumor activities of liver X receptor in pancreatic cancers

**DOI:** 10.1002/cai2.12

**Published:** 2022-06-30

**Authors:** Zhikang Chen, Xiaobo Lai, Hui Ding, Aijun Zhang, Yufei Sun, Jianhua Ling, Paul J. Chiao, Zihua Chen, Xuefeng Xia

**Affiliations:** ^1^ The Hunan Provincial Key Lab of Precision Diagnosis and Treatment for Gastrointestinal Tumor Changsha Hunan China; ^2^ Department of General Surgery, Xiangya Hospital Central South University Changsha Hunan China; ^3^ International Joint Research Center of Minimally Invasive Endoscopic Technology Equipment & Standardization Changsha Hunan China; ^4^ National Clinical Research Center for Geriatric Disorders Xiangya Hospital Changsha Hunan China; ^5^ Guangzhou First People's Hospital The Second Affiliated Hospital of South China University of Technology Guangzhou Guangdong China; ^6^ Hainan Eye Hospital and Key Laboratory of Ophthalmology, Zhongshan Ophthalmic Center Sun Yat‐Sen University Haikou Hainan China; ^7^ Houston Methodist Research Institute Houston Texas USA; ^8^ Department of Molecular and Cellular Oncology The University of Texas MD Anderson Cancer Center Houston Texas USA; ^9^ GenePlus‐Beijing Institute Beijing China

**Keywords:** GW3965, liver X receptor, nuclear receptors, pancreatic cancer, signaling pathways

## Abstract

**Background:**

Limited by difficulties in early detection and availabilities of effective treatments, pancreatic cancer is a highly malignant disease with poor prognosis. Nuclear receptors are a family of ligand‐dependent transcription factors that are highly druggable therapeutic targets playing critical roles in human physiological and pathological development, including cancer. In this study, we explored the therapeutic potential as well as the molecular mechanisms of liver X receptor (LXR) agonist GW3965 in pancreatic cancer.

**Methods:**

Soft‐agar colony formation assay, xenograft tumors, Oligonucleotide microarray, Reverse transcription real‐time polymerase chain reaction, Western immunoblotting and Immunohistochemistry were used in this study.

**Results:**

We demonstrated pleotropic in vitro activities of GW3965 in pancreatic cell lines MIA PaCa‐2 and BXPC3 including reduction of cell viability, inhibition of cell proliferation, stimulation of cell death, and suppression of colony formation, which translated to significant inhibition of xenograft tumor growth *in vitro*. By mapping the gene expression profiles, we identified the up‐regulations of 188 and the down‐regulations of 92 genes common to both cell lines following GW3965 treatment. Genes responsive to GW3965 represent a variety of biological pathways vital for multiple cellular functions. Specifically, we identified that the activating transcription factor 4/thioredoxin‐interacting protein/regulated in development and DNA damage responses 1/mechanistic target of rapamycin (ATF4/TXNIP/REDD1/mTOR) signaling critically controls GW3965‐mediated regulation of cell proliferation/death. The significance of the ATF4/TXNIP/REDD1/mTOR pathway was further supported by associated expressions in xenograft tumors as well as human pancreatic cancer samples.

**Conclusions:**

This study provides the pre‐clinical evidence that LXR agonist is a promising therapy for pancreatic cancer.

## Graphical abstract

In this study, Chen's team demonstrated pleiotropic anticancer activities of liver X receptor (LXR) agonist GW3965 in pancreatic cancer, both in vitro and in vivo. They identified the critical role and clinical significance of ATF4/TXNIP/REDD1/mTOR signaling in GW3965‐induced anticancer phenotypes. These novel findings reveal that the LXR agonist is a promising therapy for pancreatic cancer.

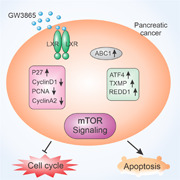

**Abbreviations**
ATF4: activating transcription factor 4
GW3965: 3‐[3‐[N‐(2‐Chloro‐3‐trifluoromethylbenzyl)‐(2,2‐diphenylethyl) amino] propyloxy] phenylacetic acid; LXR, Liver X Receptor
MAPK: 5' AMP‐activated protein kinase
mTOR: the mechanistic target of rapamycin; PI3K/Akt, phosphoinositide 3‐kinase/protein kinase B
RT‐PCR: reverse transcription real‐time polymerase chain reaction
S6K1: S6 kinase beta‐1
TXNIP: thioredoxin‐interacting protein
4EBP1: eukaryotic translation initiation factor 4E‐binding protein 1

## 1 INTRODUCTION

Pancreatic cancer is the second most common malignancy of the digestive tract and the fourth most common cause of cancer‐related deaths in the United States [1]. Substantial progress has been achieved in the understanding of the biology of tumorigenesis, which has caused earlier detection and improved therapy of most cancers. However, the incidence and mortality of pancreatic cancer are still on the rise, and approximately 80%–85% of patients present in the clinic have advanced, unresectable diseases [2]. Furthermore, pancreatic cancer is highly resistant to chemotherapy and radiation therapy and is associated with rapid growth, short median survival time, and a high death/incidence ratio even after aggressive therapy [3]. Therefore, it is important to study the molecular mechanisms underlying the occurrence and development of pancreatic cancer to identify novel targets that may benefit the diagnosis and treatment of the disease.

Liver X receptors (LXRs), members of the nuclear receptor superfamily, are pleiotropic, ligand‐activated transcription factors that function in cholesterol transport, metabolism of glucose, lipids, and carbohydrates, and inflammation [4, 5, 6, 7, 8]. In cancer, the biological activities of LXRs involve the modulation of cell proliferation, metabolism, and tumor immunity [4, 6]. Therefore, LXRs are ideal druggable targets and LXR ligands are under intensive investigation for their therapeutic potential in cancer. A previous study reported the antiproliferative effects of GW3965, a synthetic and highly selective LXR agonist [9], on multiple pancreatic cell lines [10]. However, in preclinical trials, GW3965 presented undesirable side effects of elevating plasma triglycerides and leading to steatosis in the liver [9, 11], suggesting the importance of dissecting the downstream signaling pathways mediating the effects of GW3965 to design highly specific therapeutic agents with minimal side effects.

The mechanistic target of rapamycin (mTOR) is a serine/threonine kinase that responds to multiple stimuli, including growth factors, energy, nutrients, and stress signals, regulates various downstream targets, and controls a plethora of cellular phenotypes including cell survival, proliferation, and growth [12]. The best‐characterized targets of mTOR are S6 kinase beta‐1 (S6K1) and the eukaryotic translation initiation factor 4E‐binding protein 1 (4EBP1; a repressor of messenger RNA [mRNA] translation). Through the phosphorylation and activation of S6K1 and 4EBP1, mTOR critically controls ribosome biogenesis and protein synthesis. The phosphorylation status of these two proteins is also commonly used as an indicator for mTOR activity in vitro [13]. Upstream of mTOR, several signaling molecules have been suggested to regulate mTOR activation, including phosphoinositide 3‐kinase/protein kinase B (PI3K/Akt) [14, 15], 5′ AMP‐activated protein kinase (AMPK) [16], and RAS/mitogen‐activated protein kinase (MAPK) signaling [17, 18], the choice of which seems to vary with the stimuli and cellular microenvironment. In addition, regulation in development and DNA damage responses 1 (REDD1) has been identified as a repressor of mTOR activity in response to hypoxia [19]. The expression of REDD1 is in turn controlled by AMPK [20], activating transcription factor‐4 (ATF4) on the transcriptional level [21], or by the thioredoxin‐interacting protein (TXNIP) on the protein level [22].

Although earlier studies have suggested the therapeutic potential of the LXR ligand GW3965 in targeting pancreatic cancer [10], the lack of mechanistic understanding underlying this process inhibits the design of more specific and effective treatments to further improve the benefits and minimize the side effects by GW3965. Here, we hypothesize that proteins presenting the most robust alterations in response to GW3965 are functionally important for mediating the biological effects of this ligand. To test this hypothesis, we examined the biological effects of GW3965 on pancreatic cancer, systemically characterized the gene expression profile in response to GW3965, identified the signaling mediators critical for GW3965 activities, and evaluated the clinical significance of our findings.

## 2 MATERIALS AND METHODS

### 2.1 Reagents, cell lines, experimental animals, and human samples

The following reagents were used in this study: synthetic nonsteroidal LXR agonist 3‐[3‐[*N*‐(2‐Chloro‐3‐trifluoromethylbenzyl)‐(2,2‐diphenylethyl) amino] propyloxy] phenylacetic acid (GW3965; Selleck Chemical); anti‐LXR, anti‐p27 (Abcam); goat anti‐mouse and anti‐rabbit secondary antibodies (Santa Cru); anti‐REDD1 (Protein Tech); anti‐TXNIP (MBL); anti‐mTOR, anti‐p‐mTOR, anti‐β actin, anti‐ATF4, anti‐AMPKα, anti‐p‐AMPKα, anti‐S6K1, anti‐p‐S6K1 (Cell Signaling); protein block buffer, antibody diluent solution, and EnVision+ System‐HRP (DAB) kits (Dako); and Alexa Fluor 488‐conjugated goat anti‐mouse immunoglobulin G antibody (Molecular Probes).

The pancreatic cancer cell lines MIA PaCa‐2, BXPC3, and hTERT‐immortalized but nontumorigenic human pancreatic epithelial nestin‐expressing cell line (hTERT‐HPNE) were purchased from American Type Culture Collection (ATCC) and cultured in Dulbecco's modified Eagle medium (DMEM) containing 10% (v/v) fetal bovine serum (FBS), 100 unit/ml penicillin, and 100 µg/ml streptomycin (Life Technology) at 37°C in a humidified atmosphere containing 5% CO_2_.

Four‐ to six‐week‐old male immunodeficient SCID/Beige mice were purchased from Charles River Laboratories and housed in a specific pathogen‐free facility at room temperature of 22 ± 1°C on a 12‐/12‐h light/dark cycle, with access to food and water ad libitum. The animal use and care protocols were approved by the Methodist Hospital Research Institutional Animal Care and Use Committee (Protocol number: AUP 0910‐0017).

### 2.2 Cell viability, proliferation, and apoptosis assays

Cell viability was measured using the CellTiter 96® Aqueous nonradioactive Cell Proliferation Assay (MTS) kit (Promega according to the manufacturer's instructions. Briefly, cells were seeded in 96‐well plates at a density of 2500/well and treated with either dimethyl sulfoxide (DMSO; vehicle control) or with GW3965 at different concentrations (1, 5, 10, or 20 μM) at 37°C for 48 h. The optical density of the cells was then measured with the enzyme calibration system (TECAN Safire2) at 490 nm (OD_490_). Growth inhibition was calculated as (OD_490control_ − OD_490treatment_)/OD_490control_ × 100%.

To quantify the number of viable and dead cells, cells at 48 h after treatment were stained with trypan blue (Life Technologies) and counted using a hemocytometer.

### 2.3 Soft‐agar colony formation assay

The soft‐agar colony formation assay was performed using the cell transformation detection assay kit (Cat: ECM570; EMD Millipore) according to the manufacturer's instructions. Briefly, cells were seeded in six‐well plates at a density of 2500/well in 0.4% agar/growth media over a 0.8% bottom agar layer and allowed to form colonies at 37°C in a humidified atmosphere containing 5% CO_2_. During the colony formation, the medium was exchanged every 3 days with fresh medium containing either DMSO or GW3965. Once colonies in the DMSO‐treated wells comprised at least 50 cells, all plates were stained with the cell stain solution from the kit, incubated overnight at 37°C, and counted manually.

### 2.4 Establishment of xenograft tumors

To establish xenograft tumors, 5 × 10^6^ BXPC3 or MIApaca2 cells were suspended in 0.1 ml of phosphate‐buffered saline: Matrigel (1:1) (BD Bioscience) and subcutaneously injected into nude mice. After 2 weeks, tumor size reaches an average of 100 mm^3^ (Day 0). Starting from Day 0, the mice bearing BXPC3‐ or MIApaca2‐derived tumors were randomly divided into two groups (*N* = 10 per group), with one group receiving oral gavage of GW3965 at 40 mg/kg body weight in sesame oil every other day and the other receiving DMSO in equivalent volume to GW3965 diluted in sesame oil on the same schedule. The length (*L*) and width (*W*) of the tumor were measured daily with the volume (*V*) calculated as *V* = 0.5 × *L* × *W* ^2^ [23]. On Day 21 (for MIApaca2‐inoculated mice) or Day 33 (for BXPC3‐inoculated mice), mice were killed, with the tumors isolated, weighed, and processed for further analysis.

### 2.5 Oligonucleotide microarray analysis

Gene expression analysis was performed using Affymetrix MG‐U74A (v2) GeneChips as previously described [24, 25, 26]. Briefly, total RNA was extracted from BXPC3 or MIApaca2 after 48 h‐treatment with either DMSO or GW3965 (10 µM) and reverse transcribed into complementary DNA (cDNA). Using the cDNA as the template, biotinylated cRNA was synthesized, fragmented, hybridized to the MG‐U74A GeneChips, and detected according to the manufacturer's instructions. The comparison between the treatment and control samples was performed with the Affymetrix statistical algorithm using default parameters and further analyzed using the Microsoft Access XP. Genes presenting at least two‐fold differences between the treatment and control samples were considered significantly changed.

### 2.6 Transfection of cells with small interfering RNA (siRNA)

All siRNAs used in this study, including the control nontargeting siRNA (Ctrl‐siRNA) were purchased from Dharmacon and transfected into cells using Lipofectamine RNAiMAX (Invitrogen) according to the manufacturer's instructions. At 72 h after the transfection, cells were collected for further assays.

### 2.7 Reverse transcription real‐time polymerase chain reaction (RT‐PCR)

Total RNA was extracted from the different cell lines or xenograft tumor samples using the RNeasy Mini Kit (Qiagen) according to the manufacturer's instructions. cDNA synthesis was then performed following the instructions of the TaqMan® Reverse Transcription Reagents Kit (Roche). The quantitative RT‐PCR was carried out on a Light Cycler (Roche) using Taq Man® Gene Expression Master Mix (Roche) as described previously [26]. All data were analyzed using the standard curve method. The expression of a specific target gene was examined in triplicates and normalized to that of the housekeeping gene peptidylprolyl isomerase A (PPIA).

### 2.8 Western immunoblotting

To extract total proteins, cells were lysed in buffer containing 5 0 mM Tris–HCl (pH 8.0), 150 mM NaCl, 1% NP40, 0.1% sodium dodecyl sulfate (SDS), 0.5% sodium deoxycholate, and protease inhibitors (Roche). The cell lysate was then vortexed and sonicated for 1 min on ice. The supernatants were collected after centrifugation at 12,000*g*, 4°C for 30 min and protein concentration was determined with the Bradford reagent (Bio‐Rad) using bovine serum albumin as a standard. Equal amounts of total protein were resolved and separated on 10%–12% SDS‐polyacrylamide gel electrophoresis gels, transferred onto nitrocellulose membranes (GE Healthcare) and probed with corresponding primary antibodies for 1 h at room temperature. Then, blots were incubated for 1 h with horseradish peroxidase‐conjugated anti‐rabbit immunoglobulin G (IgG) or anti‐mouse IgG (1:2000), and the signals were detected using an enhanced chemiluminescence kit (Roche). β‐Actin was used as an internal control. The band density was measured using Bio‐Rad Quantity One software.

### 2.9 Immunohistochemistry

The xenograft tumor tissues were fixed in 10% neutral‐buffered formalin, embedded into paraffin, and cut into 4‐μM‐thick sections. The slides containing xenograft tumor sections as well as those containing tissue arrays were deparaffinized in xylene and rehydrated through increasingly diluted ethanol solutions. After incubating with the peroxidase block buffer (Dako) for 15 min and steaming for 20 min in sodium citrate buffer (pH 6.0, for antigen retrieval), sections were blocked with the protein block buffer (Dako), stained with the appropriate primary antibody at 4°C in a humidified incubator overnight, and the signal was detected using the diaminobenzidine peroxidase substrate kit (Dako) according to the manufacturer's instructions. The sections were then counterstained with hematoxylin, rehydrated, mounted, and imaged using a NIKON eclipse 90i or Ti microscope. To quantify the staining signals, total staining was scored as the product of the staining intensity (on a scale of 0–3: negative = 0, weak = 1, moderate = 2, and strong = 3) and the percentage of cells showing positive staining (recorded on an ordered categorical scale: 0 = 0%, 1 = 1%–25%, 2 = 26%–50%, and 3 = 51%–100%), resulting in a scale of 0–9. The scoring was performed by two independent investigators blind to the sample information. Terminal deoxynucleotidyl transferase (TdT) dUTP nick‐end labeling (TUNEL) assay was performed on xenografts using in situ cell death detection kit, TMR red (Roche).

### 2.10 Statistical analysis

To compare the expressions of ATF4, REDD1, and TXNIP on the mRNA levels between normal pancreatic tissues and pancreatic cancerous tissues, we obtained data from the Oncomine database (https://www.oncomine.org/). Data are presented as mean ± SD. All in vitro experiments were repeated at least three times. Comparisons between two groups were performed using the Student's *t* test by IBM SPSS Statistics version 22.0. The expression of LXRα/β, Redd1, TXNIP, and mTOR in pancreatic cancer samples was analyzed by the *χ* ^2^ test by IBM SPSS Statistics version 22.0. *p* < 0.05 was considered statistically significant.

## 3 RESULTS

### 3.1 The LXR agonist, GW3965, reduced viability, inhibited proliferation, stimulated cell death, and decreased colony formation of pancreatic cancer cells *in vitro*

**Figure 1:**
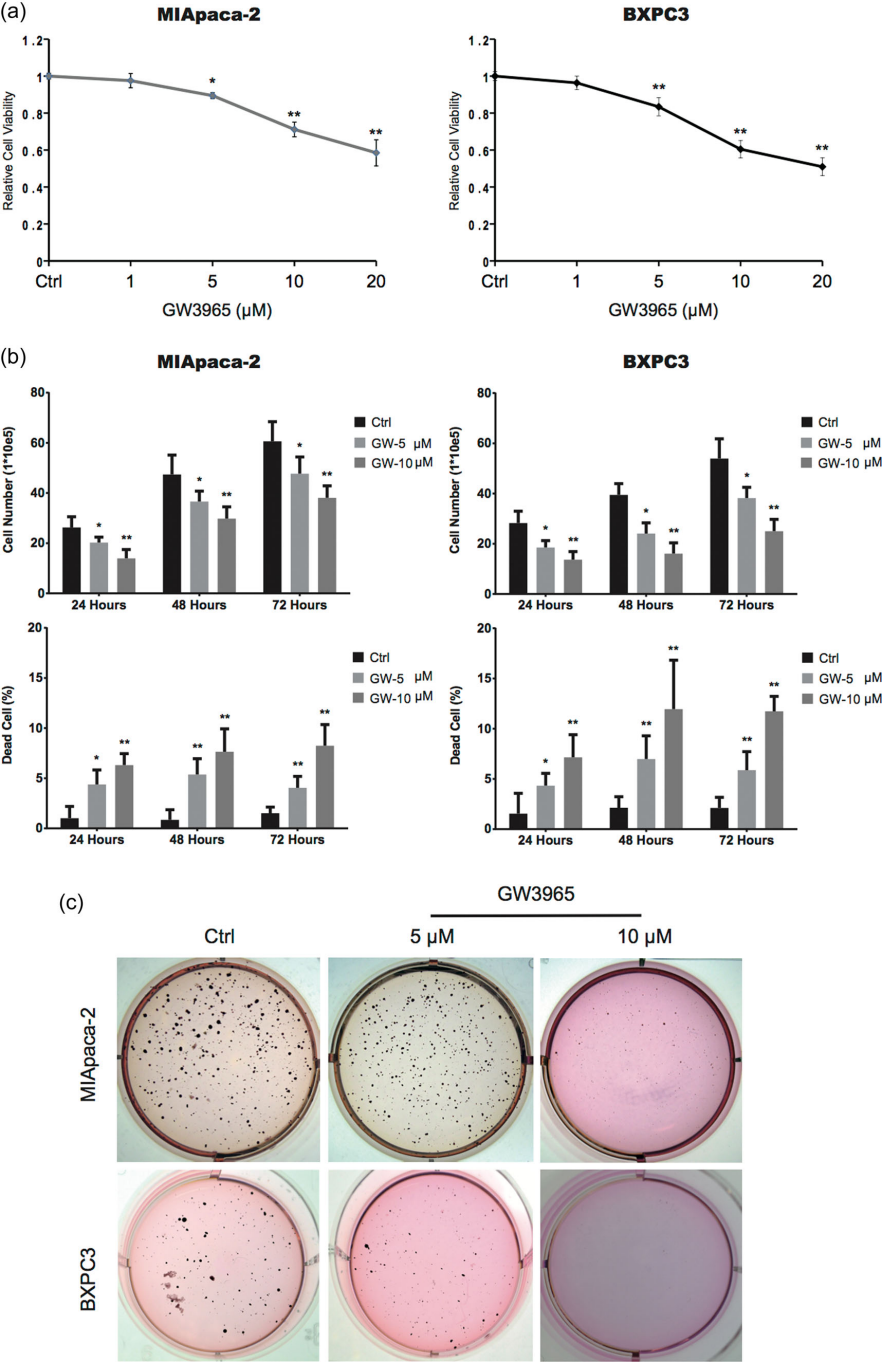
GW3965 reduced viability, inhibited proliferation, stimulated death, and decreased the colony formation of pancreatic cancer cells in vitro. (a) MIA PaCa‐2 and BXPC3 cells were treated with either dimethyl sulfoxide (Ctrl) or indicated doses of GW3965 for 48 h. Cell viability was measured and presented as a ratio to that of Ctrl cells. (b) MIA PaCa‐2 and BXPC3 cells were treated with DMSO (Ctrl), 5 or 10 μM GW3965 for indicated time periods, stained with trypan blue, and quantified using a hemocytometer. The number of trypan blue‐negative live cells and the percentage of trypan blue‐positive dead cells were compared between different treatments. (c) The transformation capability of MIA PaCa‐2 and BXPC3 cells under the treatment of DMSO (Ctrl), 5 or 10 μM GW3965 were measured by soft agar colony formation assay, with representative images of colonies grown on the plate from each group presented. *p < 0.05; **p < 0.01, when compared to the corresponding Ctrl cells.

To assess the biological activities of GW3965 on pancreatic cancer cells in general but not specific to a single cell line, we focused on two different human pancreatic cancer cell lines, MIA PaCa‐2 and BXPC3. These two cell lines are derived from two patients with pancreatic adenocarcinoma, are associated with distinct genotypes and malignant phenotypes [27], and are commonly used in studies on mechanisms of pancreatic cancer or screening for anticancer drugs. After GW3965 treatment for 48 h, the viability of both cell lines decreased in a dose‐dependent manner, with significant reductions induced by 5 μM of GW3965 (*p* < 0.05 for MIA PaCa‐2 and <0.01 for BXPC3, compared with the corresponding vehicle (DMSO)‐treated control cells; Figure 1a). The time‐course analysis on both cells treated with 5 and 10 μM GW3965 and stained with trypan blue showed that the number of trypan‐blue‐negative viable cells, although still greater with time, were significantly decreased, yet the number of trypan‐blue‐positive dead cells dramatically increased starting from 24 h after treatment (*p* < 0.05, compared with vehicle‐treated cells at the same time point; Figure 1b), suggesting that GW3965 not only inhibits the proliferation but also promotes the death of MIA PaCa‐2 and BXPC3 cells.

To analyze the effect of GW3965 on cellular transformation, we performed the soft agar colony formation assay. As shown in Figure 1c, treatment of both cell lines with 5 or 10 μM GW3965 significantly reduced the number of colonies formed.

### 3.2 GW3965 suppressed xenograft tumor growth *in vitro*

**Figure 2:**
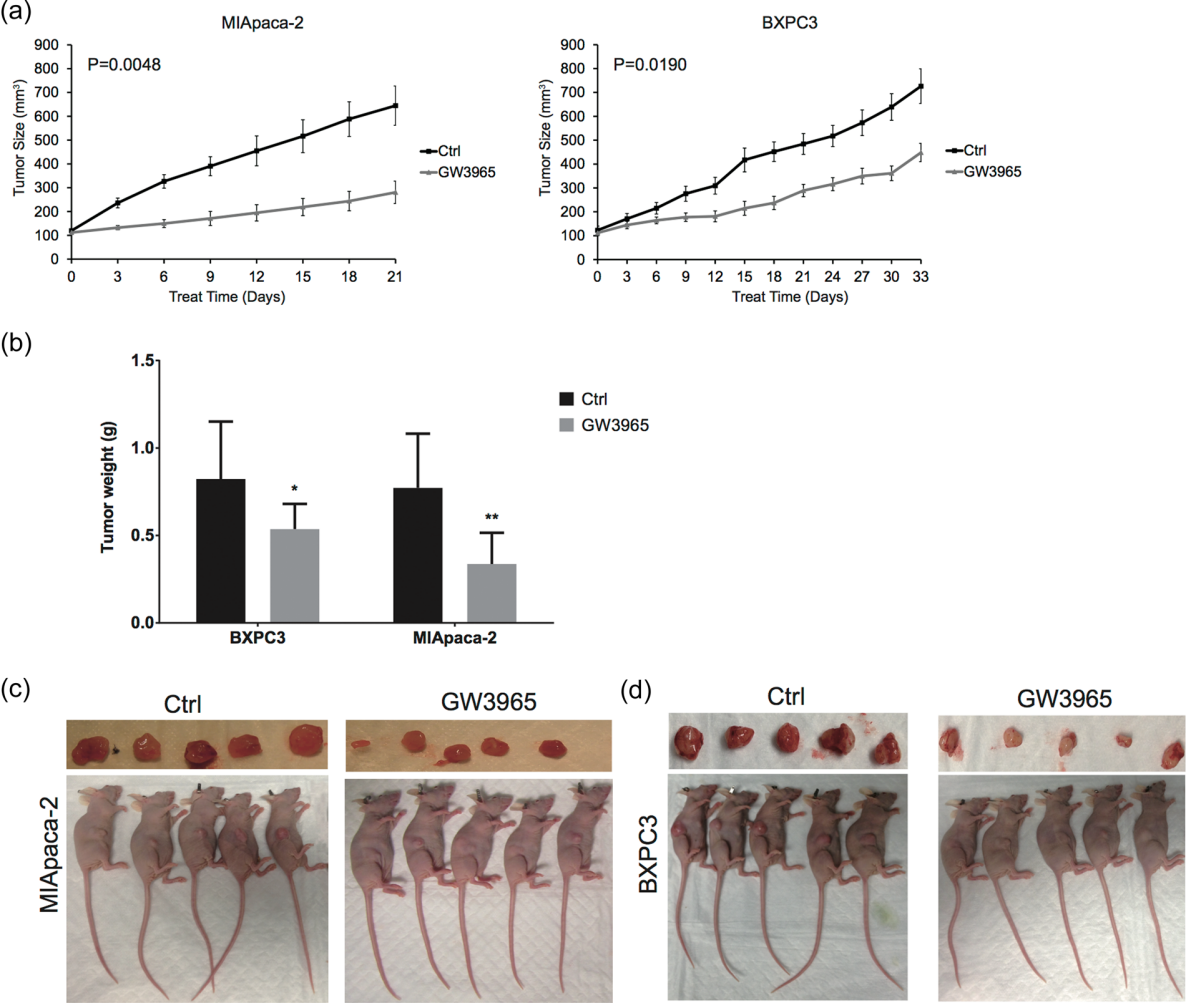
GW3965 suppressed xenograft tumor growth in vivo. Xenograft tumors were established in mice by subcutaneous injection of MIA PaCa‐2 or BXPC3 cells. (a) Following treatment with either dimethyl sulfoxide (DMSO) (Ctrl) or GW3965, the tumor size was monitored and compared between the two groups (*N* = 5/group). (b) When mice were killed on Day 21 (for MIA PaCa‐2‐inoculated mice) or Day 33 (for BXPC3‐inoculated mice) following the initiation of treatment, tumors were isolated and weighed. *p < 0.05; **p < 0.01, when compared to the corresponding Ctrl cells. (c, d) Images of mice and tumor samples.

Next, we established xenograft tumors using both MIA PaCa‐2 and BXPC3 cells to examine whether the in vitro effects of GW3965 also translate in vivo (Figure 2b,c). From 10 days (Day 0 in the graphs) after cancer cell inoculation, when the tumors from both cell lines were approximately 100 mm^3^ in volume, the mice were treated with either vehicle control (DMSO) or GW3694 (40 mg/kg body weight) (*N* = 10/group) by oral gavage every other day. As shown in Figure 2a, GW3965 significantly suppressed tumor growth from both cell lines: MIA PaCa‐2 xenograft tumors: 280.6 ± 47.1 mm^3^ for GW3965‐treated versus 644.6 ± 82.4 mm^3^ for control MIA PaCa‐2 xenograft tumors (*p* = 0.0048) on Day 21 (for MIApaca2‐inoculated mice); 448.4 ± 38.4 mm^3^ for GW3965‐treated versus 726.3 ± 72.4 mm^3^ for control BXPC3 xenograft tumors (*p* = 0.0190) on Day 33 (for BXPC3BXPC3‐inoculated mice). When the mice inoculated with MIA PaCa‐2 cells were euthanized on Day 21 and those with BXPC3 cells on Day 33 after treatment, the tumor weights and sizes were significantly lower in GW3965‐treated mice than in vehicle‐treated mice (*p* < 0.01 for MIA PaCa‐2‐inculcated mice and *p* < 0.05 for BXPC3‐inoculated mice; Figure 2b,c), indicating that GW3965 inhibits tumor growth in vivo. After dissecting the tumors, the average body weight from Ctrl and GW3965‐treated mice (for both MIA PaCa‐2‐ and BXPC3‐inoculated mice) were not significantly different from each other (*p* > 0.05; data not shown), suggesting that GW3965 treatment is not biologically safe.

### 3.3 GW3965 treatment altered the expression of various genes in multiple signaling pathways

**Figure 3:**
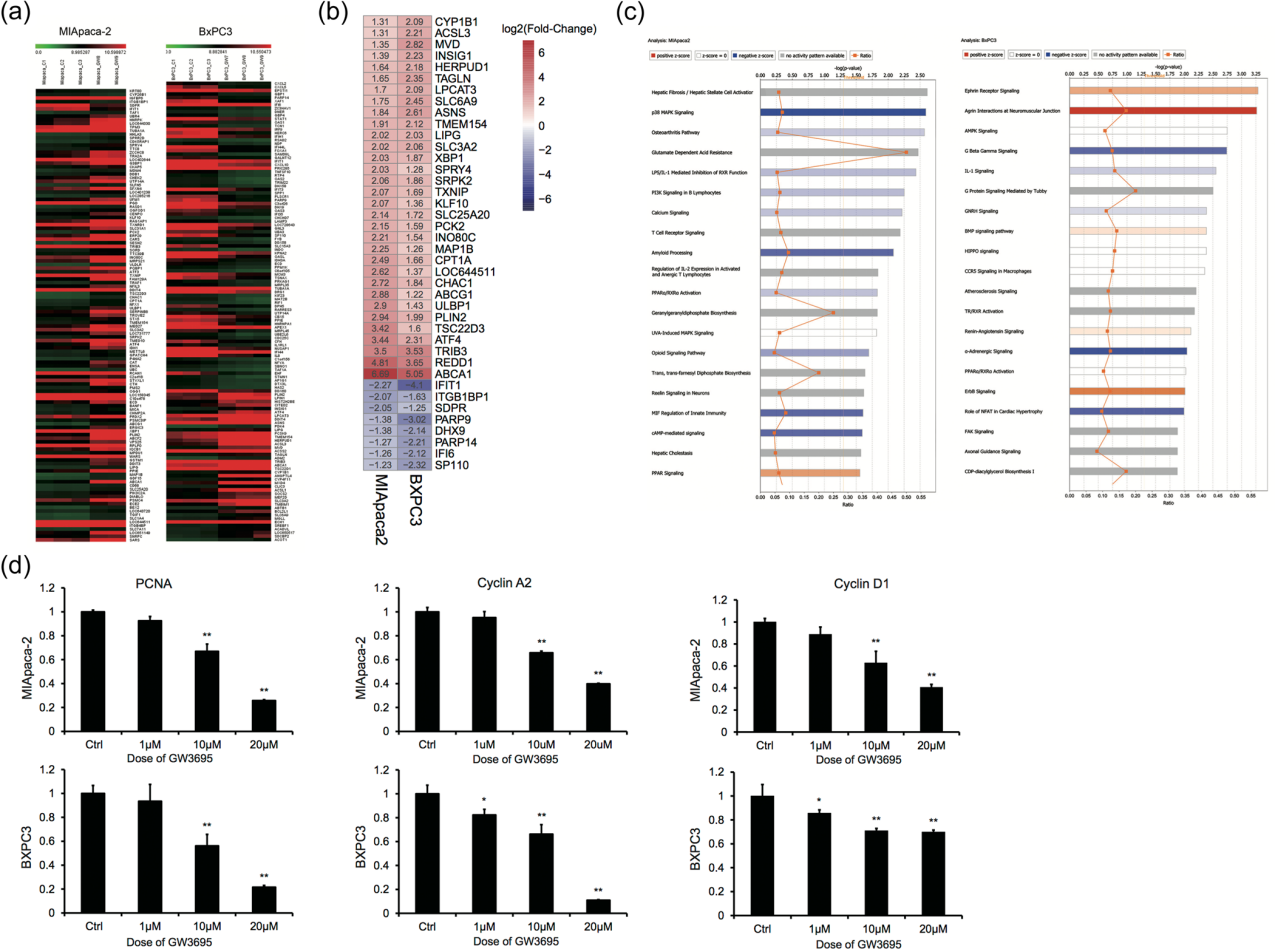
GW3965 treatment altered the expression of various genes in multiple signaling pathways. Microarray analysis was performed on MIA PaCa‐2 or BXPC3 cells following treatment with either dimethyl sulfoxide (DMSO) (Ctrl) or GW3965 and the relative expression of genes was calculated and presented as a ratio of expression in GW3965‐treated cells to that of Ctrl cells. (a, b) The heat map of 188 upregulated and 92 downregulated genes common to both pancreatic cancer cells from three representative Ctrl samples and three representative samples treated by GW3965. (c) The top‐20 signaling pathways (from top to bottom) that contained the highest number of genes with altered expressions following GW3965 treatment. (d) Quantitative real‐time polymerase chain reaction analysis on proliferating cell nuclear antigen (PCN)A, cyclin A2, and cyclin D1 in MIA PaCa‐2 (upper panels) or BXPC3 (lower panels) cells treated with DMSO (Ctrl) or indicated doses of GW3965. *p < 0.05; **p < 0.01, when compared to the corresponding Ctrl cells.

To understand the molecular mechanisms underlying the in vitro effects of GW3965, we treated MIA PaCa‐2 and BXPC3 cells with 10 μM GW3965 for 24 h, the dose and time point enabling significant alterations in cell proliferation/death from vehicle treatment (Figure 1b), and compared the gene expression profiles with DMSO‐treated control cells by microarray. A total of 755 genes were upregulated and 766 downregulated in MIA PaCa‐2 cells, while 791 were upregulated and 785 were downregulated in BXPC3 cells following GW3965 treatment. As GW3965 generated similar functional phenotypes in both cell lines, we focused on the genes altered in the same way in both cells, which included 188 upregulated and 92 downregulated genes (Figure 3a,b). These genes were distributed across multiple biological pathways, with the top 20 affected pathways centering on the regulation of DNA damage, cell cycle, steroid biosynthesis, and cancer‐related signaling (Figure 3c). In line with the inhibition of cell proliferation by GW3965, we detected a significant reduction in the steady‐state mRNA level of several genes critical for cell proliferation, including proliferating cell nuclear antigen (PCNA), cyclin A2, and cyclin D1 (Figure 3d).

### 3.4 GW3965 inhibited mTOR signaling through upregulation of mTOR inhibitor REDD1, which, in turn, was controlled by upregulation of ATF4 and TXNIP but was independent of AMPK activation

By analyzing the top 10 altered genes in both cell lines (Table 1), we identified that the cholesterol transporter ATP‐binding cassette subfamily A member 1 (ABCA1), REDD1, TRIB3, and ATF4 were among the most upregulated genes common for both MIA PaCa‐2 and BXPC3 cells in response to GW3965.

**Table 1:** The list of the top 10 genes altered in MIA PaCa‐2  (left two columns) and BXPC3 cells (right two columns) following GW3965 treatment as determined by microarray analysis

Up	Down	Up	Down
ABCA1	IFIT1	ABCA1	IFIT3
TSC22D3	KRT80	Redd1	IFIT1
Redd1	ITGB1BP1	TRIB3	PARP9
TRIB3	SDRP	ATF4	IL8
ATF4	C1QTNF6	ASNS	PLSCR1
PLIN2	STEAP3	MXD4	TUBA1A
TUBA1A	CEACAM1	MVD	ECD
PCK2	C10	SLC6A9	APEX1
ULBP1	LEAP2	TMEM154	PARP14
CHAC1	TROAP	ACADVL	UTP14A

*Note*: Up denotes upregulated genes. Down denotes downregulated genes. Fold denotes fold change in gene expression from GW3965‐treated cells relative to that from dimethyl sulfoxide‐treated cells.

**Figure 4:**
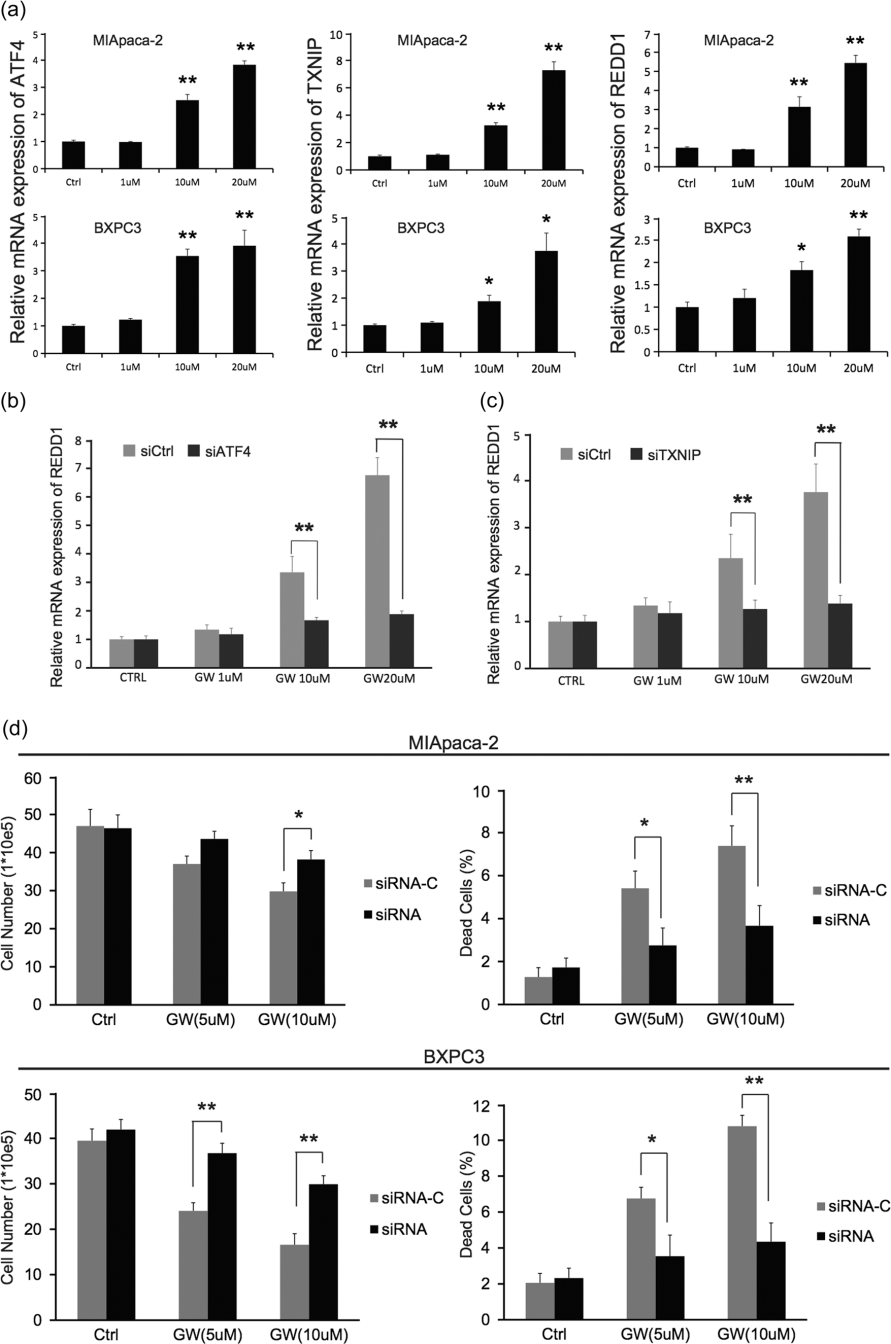
GW3965 inhibited mTOR signaling through the upregulation of mTOR inhibitor REDD1, which, in turn, was controlled by the upregulation of ATF4 and TXNIP, but independent of AMPK activation. (a) MIA PaCa‐2 and BXPC3 cells were treated with indicated doses of GW3965 for 48 h. The expression of REDD1, ATF4, and TXNIP was determined by quantitative RT‐PCR. (b) MIA PaCa‐2 and BXPC3 cells were transfected with either control (Ctrl) siRNA or ATF4 siRNA and treated with or without 10 μM GW3965 for 48 h. The expression of REDD1 was determined by quantitative RT‐PCR. (c) MIA PaCa‐2 and BXPC3 cells were transfected with either control (Ctrl) siRNA or TXNIP siRNA and treated without or with 10 μM GW3965 for 48 h. The expression of REDD1 was determined by quantitative RT‐PCR. (d) MIA PaCa‐2 and BXPC3 cells were transfected with either control (Ctrl) siRNA or REDD1 siRNA and treated without or with indicated doses GW3965 for 48 h. The cells were then stained with trypan blue and quantified using a hemocytometer. The number of trypan blue‐negative live cells and the percentage of trypan blue‐positive dead cells were compared between different treatments. AMPK, 5′ AMP‐activated protein kinase; mTOR, mechanistic target of rapamycin; RT‐PCR, real‐time polymerase chain reaction; siRNA, small interfering RNA. *p < 0.05; **p < 0.01, when compared to the corresponding Ctrl cells.

The well‐demonstrated role of mTOR signaling in diverse human cancers [12] and its regulation controlled by ATF4‐induced TNXIP‐stabilized REDD1 [21, 22, 28, 29] prompted us to examine the potential involvement of ATF4/TXNIP/REDD1/mTOR signaling in GW3965‐induced biological activities in pancreatic cancer cells. In response to treatment with GW3965 at increasing doses of MIA PaCa‐2BXPC3, we found upregulations of REDD1, ATF4, and TXNIP (Figure 4a). Furthermore, when knocking down ATF4 (Figure 4b) or TXNIP (Figure 4c) through siRNA‐induced gene silencing, the REDD1 level was not significantly affected by GW3965, suggesting that GW3965‐induced REDD1 upregulation is mediated through ATF4 and TXNIP. Functionally, knocking down REDD1 by siRNA (Supporting Information: Figure S1) significantly enhanced cell proliferation and inhibited cell death in response to GW3965 treatment (Figure 4d). To examine the specificity of ATF4 and TXNIP on GW3965‐induced REDD1 upregulation, we focused on 5′ AMP‐activated protein kinase (AMPK), a protein kinase known to regulate REDD1‐mediated inhibition on mTOR signaling in response to hypoxia‐induced energy stress [20]. We found that neither the level of AMPK nor its activation (p‐AMPK) significantly changed following GW3965 treatment in either MIA PaCa‐2 or BXPC3 cells (Supporting Information: Figure S2A). Furthermore, downregulating AMPK by siRNA did not significantly affect the REDD1 level in response to GW3965 (Supporting Information: Figure S2B), suggesting that GW3694‐induced upregulation of REDD1 is specific through ATF4 and TXNIP, but not through AMPK.

### 3.5 GW3965 led to alterations in the ATF4/TXNIP/REDD1/mTOR signaling in xenograft tumors

**Figure 5:**
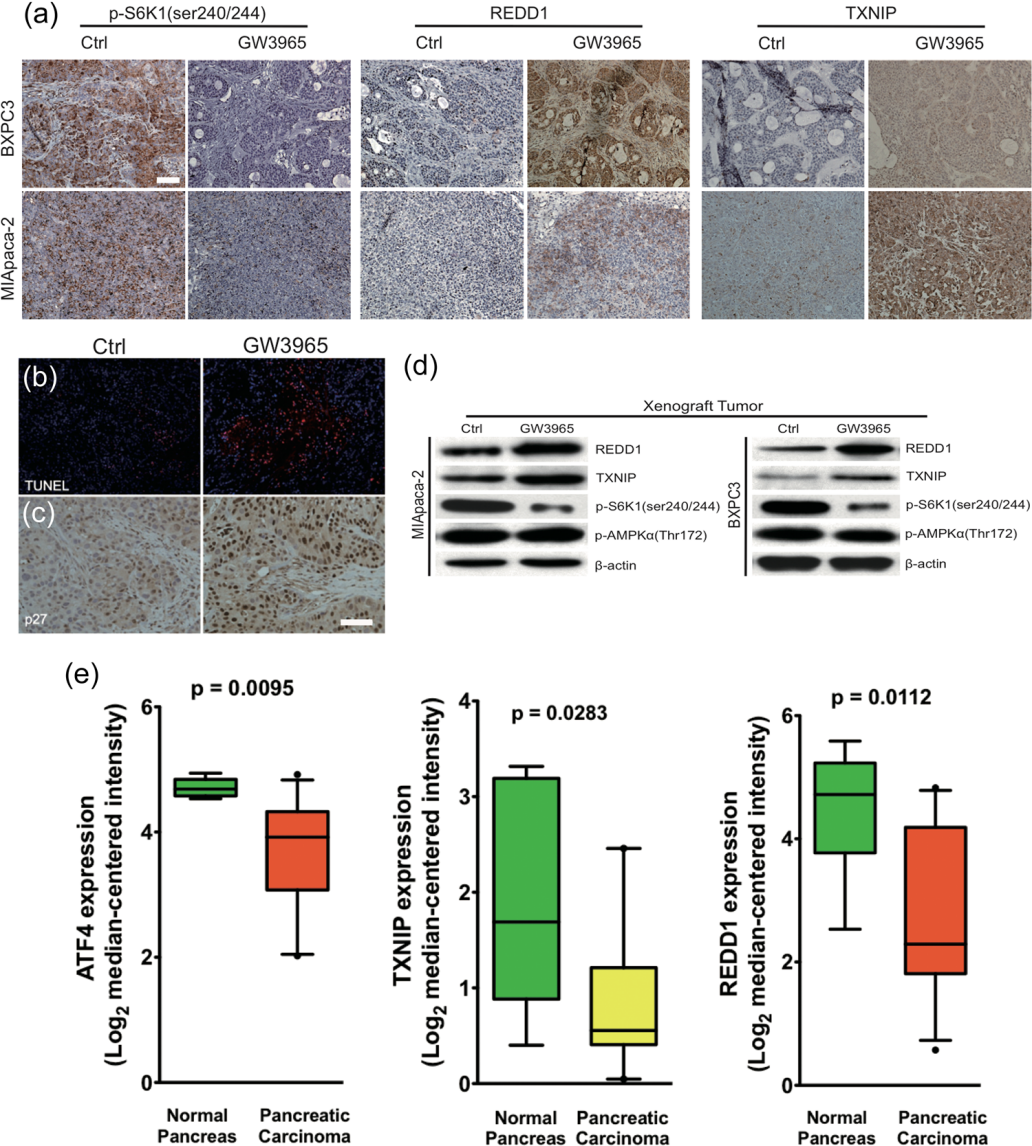
Alterations in the ATF4/TXNIP/REDD1/mTOR signaling are associated with GW3965 treatment in xenograft tumors as well as in human pancreatic cancers. Xenograft tumors derived from BXPC3 (upper panels) or MIA PaCa‐2 (lower panels) cells were isolated after treatment with DMSO (Ctrl) or GW3965. (a) The expressions of S6K1, REDD1, and TXNIP were determined by immunohistochemistry. Scale bar: 100 µm. (b, c) The status of apoptosis and cell cycle progression was measured by TUNEL immunofluorescence (red signals, b) and immunohistochemical staining of p27 (c), respectively. Scale bar: 100 µm. (d) The expressions of REDD1, TXNIP, S6K1, and p‐AMPKα (Th172) were examined by Western blot, with β‐actin detected as the internal control. (e) The expressions of REDD1, ATF4, and TXNIP from pancreatic cancer tissue and matched normal pancreatic tissue were obtained from the Oncomine database and compared between the two groups. DMSO, dimethyl sulfoxide; mTOR, mechanistic target of rapamycin; TUNEL, terminal deoxynucleotidyl transferase (TdT) dUTP nick‐end labeling.

To explore whether the ATF4/TXNIP/REDD1/mTOR signaling may also play a role in GW3965 activity *in vivo*, we compared the expressions of these proteins between vehicle‐treated and GW‐3695‐treated xenograft tumors by immunohistochemistry (Figure 5a) as well as Western immunoblot (Figure 5d), which showed the downregulation of p‐S6K1, the upregulation of REDD1 and TXNIP, and a nonsignificant change of p‐AMPK, consistent with the findings from the in vitro cell lines. To assess the biological effects of GW3695 *in vivo*, we performed a TUNEL assay (to assess apoptosis) and IHC staining for p27 (a cyclin‐dependent kinase inhibitor that inhibits G1 cell‐cycle progression) in xenografts from Ctrl and GW3695‐treated mice. As shown in Figure 5b,c both TUNEL and P27 signals were significantly higher in GW3695 xenografts, suggesting that GW3695 not only significantly inhibits cell‐cycle progression, but also potently induces apoptosis in vivo.

### 3.6 Human pancreatic cancer was associated with alterations in the ATF4/TXNIP/REDD1/mTOR signaling

To assess the clinical relevance of our observations, we first compared the mRNA expressions of ATF4, TXNIP, and REDD1 on the basal level between immortalized but nontumorigenic HPNE cells and pancreatic cancer cells. We found that all three molecules were significantly downregulated in the cancer cells, supporting their associations with pancreatic cancers (Supporting Information: Figure S3). Furthermore, we performed an analysis of the mRNA expressions on REDD1, ATF4, and TXNIP from pancreatic cancer samples and the matching normal pancreatic tissues (the Oncomine database; Figure 5e). We found significantly reduced levels of all three proteins in the cancer tissues when compared to the matching normal pancreatic tissues (*p* = 0.0112 for REDD1, 0.0095 for ATF4, and 0.0283 for TXNIP).

## 4 DISCUSSION

In this study, we demonstrated the antiproliferative and pro‐death effects of the LXR agonist GW3965 on pancreatic cells in vitro and the inhibition of xenograft tumor growth in vivo. We also revealed the gene expression profiles and biological pathways altered in response to GW3965. Most importantly, we uncovered the significance of ATF4/TXNIP/REDD1/mTOR signaling in the control of the biological activities of GW3965, which was corroborated by expressional analysis on xenograft tumor samples as well as human tissues of pancreatic cancer. To our knowledge, this is the first report showing the importance of ATF4/TXNIP/REDD1/mTOR signaling in the anticancer activity of LXR agonists.

Dysregulation of metabolism is closely associated with the development of cancer. As a key regulator in the metabolism of glucose, cholesterol, lipid, and carbohydrate, the first evidence suggestive of LXR involvement in cancer came from studies demonstrating upregulation of LXR target gene sterol response element‐binding protein 1c (SREBP1c) in prostate cancer [30, 31]. Motivated by these observations, Fukuchi et al. [4] investigated the effects of LXR synthetic agonist T0901317 in prostate cancer cells and demonstrated its antiproliferative and antitumor effects. Since then, the anticancer activities of LXR agonists, T0901317 or GW3965 in most cases, have been reported in multiple cancers, including prostate, breast, ovarian, skin, pancreas, brain, lung, colon, and blood cancers [32]. Mechanistic studies have identified various target genes of LXR agonists that collectively modulate four major phenotypes: cell cycle, metabolism, hormone signaling, and immune regulation [32].

Among the targets for cell cycle regulation, p27, an inhibitor for cyclin‐dependent kinase (CDK), is upregulated in response to LXR agonists in prostate cancer [4], ovarian cancer [33], but not in breast cancer [34] or leukemia [35], suggesting the differential regulations of the cell‐cycle machinery in different cell types by LXR signaling. In this study, we observed the antiproliferative and pro‐death activities of GW3965 in two pancreatic cell lines, MIA PaCa‐2 and BXPC3, consistent with the previous study [10]. Mechanistically, we uncovered the downregulation of PCNA, cyclin A2, and cyclin D1, consistent with GW3965‐induced antiproliferation. In addition, several cell‐cycle‐related biological pathways, including p53 signaling, G2/M DNA damage checkpoint regulation, checkpoint kinase (CHK) proteins in cell‐cycle checkpoint control, chromosomal replication, mitotic roles of polo‐like kinase, aminoacyl‐tRNA biosynthesis, and cyclins and cell‐cycle regulation, were among the most significantly altered pathways in both pancreatic cell lines following GW3965 treatment, corroborating the importance of LXR agonists in cell‐cycle modulation in pancreatic cancer.

Of the metabolic targets of LXR agonists in cancer, ABCA1 is the best characterized, the reduced expression of which is associated with prostate carcinogenesis [36] and its upregulation is induced by LXR agonists. In addition to regulating cholesterol transport, genes involved in lipid metabolism, including lipogenic targets SREBP1, fatty acid synthase (FASN) [37], low‐density lipoprotein receptor (LDLR) [38], and apolipoprotein E (APOE), are also suggested to contribute to LXR‐mediated antiproliferation and proapoptosis in different cancer types. Consistently, we identified ABCA1 as a robustly upregulated gene in both MIA PaCa‐2 and BXPC3 cells following GW3965 treatment, suggesting that the regulation of cholesterol transport is a mechanism by which GW3965 controls cell proliferation in pancreatic cancer.

In hormone‐dependent cancers, such as prostate and breast cancer, LXR also acts by regulating hormone metabolism, such as antagonism of androgen receptor signaling [39], upregulation of sulphotransferase 2A1, and downregulation of steroid sulphatase to inactivate androgen in prostate cancer [40], and reduction of the expression of estrogen receptor α in breast cancer [34, 41]. Consistent with the importance of the pancreas as an endocrine and exocrine gland, we showed that the biosynthesis of steroids was significantly altered in pancreatic cancer cells in response to GW3965 treatment, supporting the involvement of hormone regulation in pancreatic cancer development and its potential for therapy.

In addition to directly regulating cancer cells, LXR agonists are also shown to modulate the behaviors of immune cells and other mesenchymal cells within the tumor microenvironment. For dendritic cells, T0901317 suppresses the expression of chemokine receptor CCR7, preventing the migration of these cells to lymph nodes, and inhibiting dendritic cell‐mediated antitumor immunity [42]. In contrast, the LXR ligand stimulates the production of pro‐inflammatory cytokine interferon γ (IFNγ) from macrophages and T cells, promoting immune surveillance [43]. LXR also stimulates the secretion of APOE from macrophages and other stromal cells to inhibit tumor growth, angiogenesis, or metastasis [44, 45]. On vascular endothelial cells, LXR agonists inhibit signaling from vascular endothelial growth factor receptor 2 (VEGFR2), reducing endothelial proliferation and vasculogenesis [46]. Although the in vitro cell systems (MIA PaCa‐2 and BXPC3) used in this study provides no information on GW3965 effects on stromal cells in pancreatic cancer, the significant inhibition of GW3965 on the growth of xenograft tumors in mice does not exclude the potential contribution of stromal regulation by the LXR agonist. It would be interesting to examine any potential alterations within the stromal components of pancreatic cancer both in experimental animals and in cancer patients.

Given that LXR is a ligand‐activated transcription factor, most efforts to understand its molecular mechanisms have been focused on the target genes presenting altered expressions in response to LXR ligands. Very few studies have revealed the effects of LXR agonists on intracellular signaling pathways and how these pathways may contribute to the biological activities of LXR agonists. In this study, through systemic microarray analysis, we not only identified 188 upregulated and 92 downregulated genes in both MIA PaCa‐2 and BXPC3 cells but also revealed the biological pathways most responsive to GW3965 treatment, including those involved in regulating DNA damage, cell cycle, steroid biosynthesis, and cancer‐related signaling. Specifically, we noticed that REDD1 and ATF4 were among the most highly upregulated genes in both pancreatic cell lines, which, together with previous studies showing the crosstalk among ATF4, REDD1, mTOR, and TXNIP [21, 22, 28], prompted us to explore the potential involvement of ATF4/TXNIP/REDD1/mTOR signaling in GW3965‐mediated antiproliferation and pro‐death of pancreatic cancer cells. The expression analysis showed that in both cells and in xenograft tumors, GW3965 treatment is associated with the downregulation of mTOR activity, as represented by p‐S6K1 expression level, and the upregulation of ATF4, TXNIP, and REDD1 levels. More importantly, analysis of human pancreatic cancer samples revealed significant downregulation of ATF4, TXNIP, and REDD1 in the tumor tissues compared to matching normal tissues. Although their downregulation could be simply a bystander event during pancreatic cancer development, our findings revealed that the upregulations of ATF4, TXNIP, and REDD1 were concomitant with the therapeutic benefits of GW3965. Further studies using siRNA‐mediated gene silencing showed that ATF4 and TXNIP were essential for GW3965‐induced REDD1 expression, while REDD1 was critical for GW3965‐mediated antiproliferation and pro‐death activities in pancreatic cancer cells. Collectively, the data support and justify future studies to look into the biological significance of these three molecules in pancreatic cancer development, among samples beyond two pancreatic cell lines. The data also indicate that in addition to regulating mTOR signaling in response to hypoxia, nutrient deprivation, and ER stress [21, 22, 47], REDD1 also controls mTOR activity in pancreatic cancers. Upregulation of REDD1 presented anticancer activities and may become a target for cancer therapy. In response to hypoxia‐induced energy stress, REDD1‐mediated inhibition of mTOR activity was dictated by AMPK [20]. In pancreatic cancer cells, however, not only was AMPK level or its activation (p‐AMPK) altered by GW3965 treatment but knocking down AMPK by siRNA did not affect REDD1 level in response to GW3965, suggesting the specificity of ATF4 and TXNIP in regulating REDD1 and mTOR in these cancer cells.

In summary, we confirmed previous findings on anticancer activities of LXR agonists. Furthermore, we identified the differentially expressed genes and biological pathways involved in the responses of pancreatic cancer cells to LXR agonists. Most importantly, we demonstrated the significance of ATF4/TXNIP/REDD1/mTOR signaling in the antiproliferation and pro‐death activities of LXR agonists. Future studies should be directed toward understanding molecular mechanisms underlying the regulation of ATF4/TXNIP/REDD1/mTOR axis by GW3965. Dissecting specific target genes or signaling pathways responsible for each component of GW3965 biological activity would benefit the design of highly specific and maximally safe cancer therapy.

## AUTHOR CONTRIBUTIONS

**Xuefeng Xia**: supervision (lead); writing – original draft (lead); writing – review & editing (lead). **Zhikang Chen**: data curation (lead); formal analysis (lead); funding acquisition (lead); investigation (lead); methodology (lead); writing – original draft (lead). **Xiaobo Lai**: data curation (equal); methodology (equal). **Hui Ding**: data curation (equal); formal analysis (equal); methodology (equal). **Aijun Zhang**: data curation (equal); formal analysis (equal); methodology (equal). **Paul Chiao**: supervision (equal). **Jianhua Lin**: data curation (equal); methodology (equal). **Yufei Sun**: formal analysis (equal). **Zihua Chen**: funding acquisition (equal); supervision (equal).

## CONFLICT OF INTEREST

The authors declare no conflict of interest.

## ETHICS STATEMENT

None.

## INFORMED CONSENT

Not applicable.

## Supporting information

This article includes online‐only Supplemental Data.

Supporting information.

Supporting information.

Supporting information.

Supporting information.

## Data Availability

I accept data availability if it is accepted for publication.

## References

[1] Siegel R , Naishadham D , Jemal A . Cancer statistics, 2013. CA Cancer J Clin. 2013;63:11–30. 10.3322/caac.21166 23335087

[2] Vincent A , Herman J , Schulick R , Hruban RH , Goggins M . Pancreatic cancer. Lancet. 2011;378:607–20. 10.1016/S0140-6736(10)62307-0 21620466 PMC3062508

[3] Raimondi S , Maisonneuve P , Lowenfels AB . Epidemiology of pancreatic cancer: an overview. Nat Rev Gastroenterol Hepatol. 2009;6:699–708. 10.1038/nrgastro.2009.177 19806144

[4] Fukuchi J , Kokontis JM , Hiipakka RA , Chuu CP , Liao S . Antiproliferative effect of liver X receptor agonists on LNCaP human prostate cancer cells. Cancer Res. 2004;64:7686–9. 10.1158/0008-5472.CAN-04-2332 15520170

[5] Hong C , Tontonoz P . Coordination of inflammation and metabolism by PPAR and LXR nuclear receptors. Curr Opin Genet Dev. 2008;18:461–7. 10.1016/j.gde.2008.07.016 18782619 PMC2641014

[6] Russo V . Metabolism, LXR/LXR ligands, and tumor immune escape. J Leukoc Biol. 2011;90:673–9. 10.1189/jlb.0411198 21771899

[7] Tontonoz P , Mangelsdorf DJ . Liver X receptor signaling pathways in cardiovascular disease. Mol Endocrinol. 2003;17:985–93. 10.1210/me.2003-0061 12690094

[8] Xiao X , Wang P , Chou KC . Recent progresses in identifying nuclear receptors and their families. Curr Top Med Chem. 2013;13:1192–200. 23647541 10.2174/15680266113139990006

[9] Collins JL , Fivush AM , Watson MA , Galardi CM , Lewis MC , Moore LB , et al. Identification of a nonsteroidal liver X receptor agonist through parallel array synthesis of tertiary amines. J Med Chem. 2002;45:1963–6. 10.2174/15680266113139990006 11985463

[10] Candelaria NR , Addanki S , Zheng J , Nguyen‐Vu T , Karaboga H , Dey P , et al. Antiproliferative effects and mechanisms of liver X receptor ligands in pancreatic ductal adenocarcinoma cells. PLOS One. 2014;9:e106289. 10.1371/journal.pone.0106289 25184494 PMC4153644

[11] Joseph SB , McKilligin E , Pei L , Watson MA , Collins AR , Laffitte BA , et al. Synthetic LXR ligand inhibits the development of atherosclerosis in mice. Proc Natl Acad Sci U S A. 2002;99:7604–9. 10.1073/pnas.112059299 12032330 PMC124297

[12] Populo H , Lopes JM , Soares P . The mTOR signalling pathway in human cancer. Int J Mol Sci. 2012;13:1886–918. 10.3390/ijms13021886 22408430 PMC3291999

[13] Hay N , Sonenberg N . Upstream and downstream of mTOR. Genes Dev. 2004;18:1926–45. 10.1101/gad.1212704 15314020

[14] LoPiccolo J , Blumenthal GM , Bernstein WB , Dennis PA . Targeting the PI3K/Akt/mTOR pathway: effective combinations and clinical considerations. Drug Resist Update. 2008;11:32–50. 10.1016/j.drup.2007.11.003 PMC2442829 18166498

[15] Vander Haar E , Lee SI , Bandhakavi S , Griffin TJ , Kim DH . Insulin signalling to mTOR mediated by the Akt/PKB substrate PRAS40. Nat Cell Biol. 2007;9:316–23. 10.1038/ncb1547 17277771

[16] Xu J , Ji J , Yan XH . Cross‐talk between AMPK and mTOR in regulating energy balance. Crit Rev Food Sci Nutr. 2012;52:373–81. 10.1080/10408398.2010.500245 22369257

[17] Ma L , Chen Z , Erdjument‐Bromage H , Tempst P , Pandolfi PP . Phosphorylation and functional inactivation of TSC2 by Erk implications for tuberous sclerosis and cancer pathogenesis. Cell. 2005;121:179–93. 10.1016/j.cell.2005.02.031 15851026

[18] Ma L , Teruya‐Feldstein J , Bonner P , Bernardi R , Franz DN , Witte D , et al. Identification of S664 TSC2 phosphorylation as a marker for extracellular signal‐regulated kinase‐mediated mTOR activation in tuberous sclerosis and human cancer. Cancer Res. 2007;67:7106–12. 10.1158/0008-5472.CAN-06-4798 17671177

[19] Brugarolas J , Lei K , Hurley RL , Manning BD , Reiling JH , Hafen E , et al. Regulation of mTOR function in response to hypoxia by REDD1 and the TSC1/TSC2 tumor suppressor complex. Genes Dev. 2004;18:2893–904. 10.1101/gad.1256804 15545625 PMC534650

[20] Schneider A , Younis RH , Gutkind JS . Hypoxia‐induced energy stress inhibits the mTOR pathway by activating an AMPK/REDD1 signaling axis in head and neck squamous cell carcinoma. Neoplasia. 2008;10:1295–302. 10.1593/neo.08586 18953439 PMC2570606

[21] Whitney ML , Jefferson LS , Kimball SR . ATF4 is necessary and sufficient for ER stress‐induced upregulation of REDD1 expression. Biochem Biophys Res Commun. 2009;379:451–5. 10.1016/j.bbrc.2008.12.079 19114033 PMC2656673

[22] Jin HO , Seo SK , Kim YS , Woo SH , Lee KH , Yi JY , et al. TXNIP potentiates REDD1‐induced mTOR suppression through stabilization of REDD1. Oncogene. 2011;30:3792–801. 10.1038/onc.2011.102 21460850

[23] Tomayko MM , Reynolds CP . Determination of subcutaneous tumor size in athymic (nude) mice. Cancer Chemother Pharmacol. 1989;24:148–54. 10.1007/BF00300234 2544306

[24] Wang Z , Malone MH , He H , McColl KS , Distelhorst CW . Microarray analysis uncovers the induction of the proapoptotic BH3‐only protein Bim in multiple models of glucocorticoid‐induced apoptosis. J Biol Chem. 2003;278:23861–7. 10.1074/jbc.M301843200 12676946

[25] Wang Z , Malone MH , Thomenius MJ , Zhong F , Xu F , Distelhorst CW . Dexamethasone‐induced gene 2 (dig2) is a novel pro‐survival stress gene induced rapidly by diverse apoptotic signals. J Biol Chem. 2003;278:27053–8. 10.1074/jbc.M303723200 12736248

[26] Zhang A , Sieglaff DH , York JP , Suh JH , Ayers SD , Winnier GE , et al. Thyroid hormone receptor regulates most genes independently of fibroblast growth factor 21 in liver. J Endocrinol. 2015;224:289–301. 10.1530/JOE-14-0440 25501997

[27] Deer EL , Gonzalez‐Hernandez J , Coursen JD , Shea JE , Ngatia J , Scaife CL , et al. Phenotype and genotype of pancreatic cancer cell lines. Pancreas. 2010;39:425–35. 10.1097/MPA.0b013e3181c15963 20418756 PMC2860631

[28] Jin HO , Seo SK , Woo SH , Kim ES , Lee HC , Yoo DH , et al. SP600125 negatively regulates the mammalian target of rapamycin via ATF4‐induced REDD1 expression. FEBS Lett. 2009;583:123–7. 10.1074/jbc.M109.047688 19059405

[29] Kimball SR , Jefferson LS . Induction of REDD1 gene expression in the liver in response to endoplasmic reticulum stress is mediated through a PERK, eIF2alpha phosphorylation, ATF4‐dependent cascade. Biochem Biophys Res Commun. 2012;427:485–9. 10.1016/j.bbrc.2012.09.074 23000413 PMC3482272

[30] Ettinger SL , Sobel R , Whitmore TG , Akbari M , Bradley DR , Gleave ME , et al. Dysregulation of sterol response element‐binding proteins and downstream effectors in prostate cancer during progression to androgen independence. Cancer Res. 2004;64:2212–21. 10.1158/0008-5472.can-2148-2 15026365

[31] Sporer A , Brill DR , Schaffner CP . Epoxycholesterols in secretions and tissues of normal, benign, and cancerous human prostate glands. Urology. 1982;20:244–50. 10.1016/0090-4295(82)90631-8 6181602

[32] Lin CY , Gustafsson JA . Targeting liver X receptors in cancer therapeutics. Nat Rev Cancer. 2015;15:216–24. 10.1038/nrc3912 25786697

[33] Rough JJ , Monroy MA , Yerrum S , Daly JM . Anti‐proliferative effect of LXR agonist T0901317 in ovarian carcinoma cells. J Ovarian Res. 2010;3:13. 10.1186/1757-2215-3-13 20504359 PMC2890636

[34] Vedin LL , Lewandowski SA , Parini P , Gustafsson JA , Steffensen KR . The oxysterol receptor LXR inhibits proliferation of human breast cancer cells. Carcinogenesis. 2009;30:575–9. 10.1093/carcin/bgp029 19168586

[35] Geyeregger R , Shehata M , Zeyda M , Kiefer FW , Stuhlmeier KM , Porpaczy E , et al. Liver X receptors interfere with cytokine‐induced proliferation and cell survival in normal and leukemic lymphocytes. J Leukoc Biol. 2009;86:1039–48. 10.1189/jlb.1008663 19671841

[36] Fukuchi J , Hiipakka RA , Kokontis JM , Hsu S , Ko AL , Fitzgerald ML , et al. Androgenic suppression of ATP‐binding cassette transporter A1 expression in LNCaP human prostate cancer cells. Cancer Res. 2004;64:7682–5. 10.1158/0008-5472.CAN-04-2647 15520169

[37] Kim KH , Lee GY , Kim JI , Ham M , Won Lee J , Kim JB . Inhibitory effect of LXR activation on cell proliferation and cell cycle progression through lipogenic activity. J Lipid Res. 2010;51:3425–33. 10.1194/jlr.M007989 20847297 PMC2975714

[38] Guo D , Reinitz F , Youssef M , Hong C , Nathanson D , Akhavan D , et al. An LXR agonist promotes glioblastoma cell death through inhibition of an EGFR/AKT/SREBP‐1/LDLR‐dependent pathway. Cancer Discov. 2011;1:442–56. 10.1158/2159-8290.CD-11-0102 22059152 PMC3207317

[39] Chuu CP , Chen RY , Hiipakka RA , Kokontis JM , Warner KV , Xiang J , et al. The liver X receptor agonist T0901317 acts as androgen receptor antagonist in human prostate cancer cells. Biochem Biophys Res Commun. 2007;357:341–6. 10.1016/j.bbrc.2007.03.116 17416342 PMC2693411

[40] Lee JH , Gong H , Khadem S , Lu Y , Gao X , Li S , et al. Androgen deprivation by activating the liver X receptor. Endocrinology. 2008;149:3778–88. 10.1210/en.2007-1605 18450964 PMC2488233

[41] Nguyen‐Vu T , Vedin LL , Liu K , Jonsson P , Lin JZ , Candelaria NR , et al. Liver X receptor ligands disrupt breast cancer cell proliferation through an E2F‐mediated mechanism. Breast Cancer Res. 2013;15:R51. 10.1186/bcr3443 23809258 PMC4053202

[42] Villablanca EJ , Raccosta L , Zhou D , Fontana R , Maggioni D , Negro A , et al. Tumor‐mediated liver X receptor‐alpha activation inhibits CC chemokine receptor‐7 expression on dendritic cells and dampens antitumor responses. Nat Med. 2010;16:98–105. 10.1038/nm.2074 20037595

[43] Wang Q , Ma X , Chen Y , Zhang L , Jiang M , Li X , et al. Identification of interferon‐gamma as a new molecular target of liver X receptor. Biochem J. 2014;459:345–54. 10.1042/BJ20131442 24438183

[44] El Roz A , Bard JM , Valin S , Huvelin JM , Nazih H . Macrophage apolipoprotein E and proliferation of MCF‐7 breast cancer cells: role of LXR. Anticancer Res. 2013;33:3783–9. 24023310

[45] Pencheva N , Buss CG , Posada J , Merghoub T , Tavazoie SF . Broad‐spectrum therapeutic suppression of metastatic melanoma through nuclear hormone receptor activation. Cell. 2014;156:986–1001. 10.1016/j.cell.2014.01.038 24581497

[46] Noghero A , Perino A , Seano G , Saglio E , Lo Sasso G , Veglio F , et al. Liver X receptor activation reduces angiogenesis by impairing lipid raft localization and signaling of vascular endothelial growth factor receptor‐2. Arterioscler Thromb Vasc Biol. 2012;32:2280–8. 10.1161/ATVBAHA.112.250621 22723445

[47] Katiyar S , Liu E , Knutzen CA , Lang ES , Lombardo CR , Sankar S , et al. REDD1, an inhibitor of mTOR signalling, is regulated by the CUL4A‐DDB1 ubiquitin ligase. EMBO Rep. 2009;10:866–72. 10.1038/embor.2009.93 19557001 PMC2726664

