# Supplementary material for: ATF4/TXNIP/REDD1/mTOR signaling mediates the antitumor activities of liver X receptor in pancreatic cancers
Source: Cancer Innov. 2022 Jun 30;1(1):55–69. doi: 10.1002/cai2.12 (PMC10686145; doi:10.1002/cai2.12)
Supplement: Supplementary file 1 — Supporting information. [file CAI2-1-55-s002.docx]

**Supporting information**

**Supplemental Figure 1. REDD1 was significantly reduced by siRNA-mediated gene silencing.** MIA PaCa-2 (left panel) and BXPC3 (right panel) were transfected with either control (Ctrl) siRNA or REDD1 siRNA. Expression of REDD1 was determined by quantitative RT-PCR.

**Supplemental Figure 2. GW3965-induced up-regulation of REDD1 was not mediated through AMPKα.** A. MIA PaCa-2 or BXPC3 cells were treated with either DMSO (Ctrl) or indicated doses of GW3965 for 48 h. The expressions of p-AMPKα (Thr172) and AMPKα were determined by Western blot, with β-actin detected as the internal control. B. MIA PaCa-2 or BXPC3 cells were transfected with either control (-) or AMPKα (+) siRNA and treated with either DMSO (Ctrl) or 10 μM GW3965 for 48 h. The expression of AMPKα and REDD1 was determined by Western blot, with β-actin detected as the internal control.

**Supplemental Figure 3.** Quantitative real-time PCR analysis on ATF4, TXNIP and REDD1 expressions in HPNE, MIA PaCa-2, and BXPC3 cells. **P* < 0.05; ***P* < 0.01, when compared to HPNE cells.
