# Supplementary figures and images for: ATF4/TXNIP/REDD1/mTOR signaling mediates the antitumor activities of liver X receptor in pancreatic cancers
Source: Cancer Innov. 2022 Jun 30;1(1):55–69. doi: 10.1002/cai2.12 (PMC10686145; doi:10.1002/cai2.12)

# Supplementary Figure 1

A.

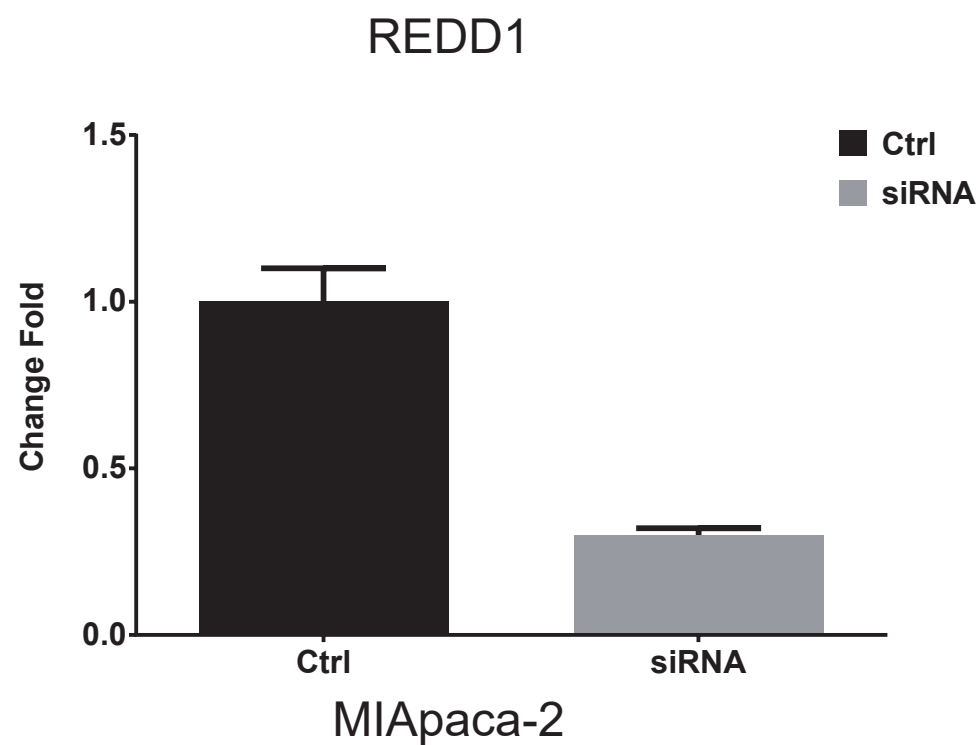

B.

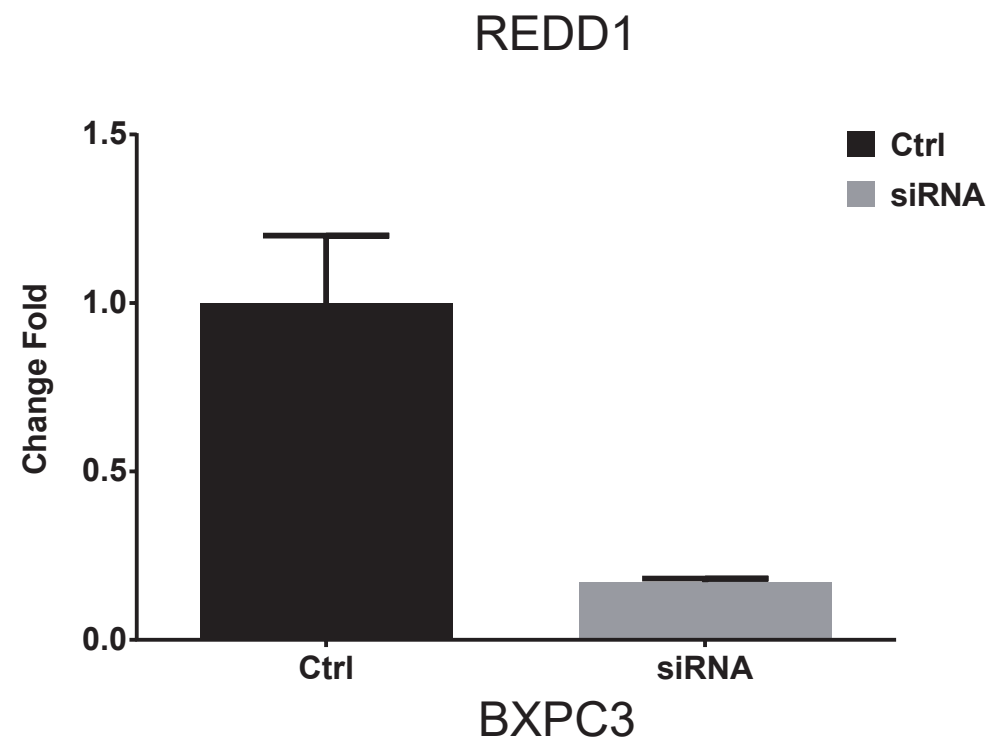

Supplement: Supplementary file 2 — Supporting information. [file CAI2-1-55-s004.pdf]

# Supplementary Figure 2

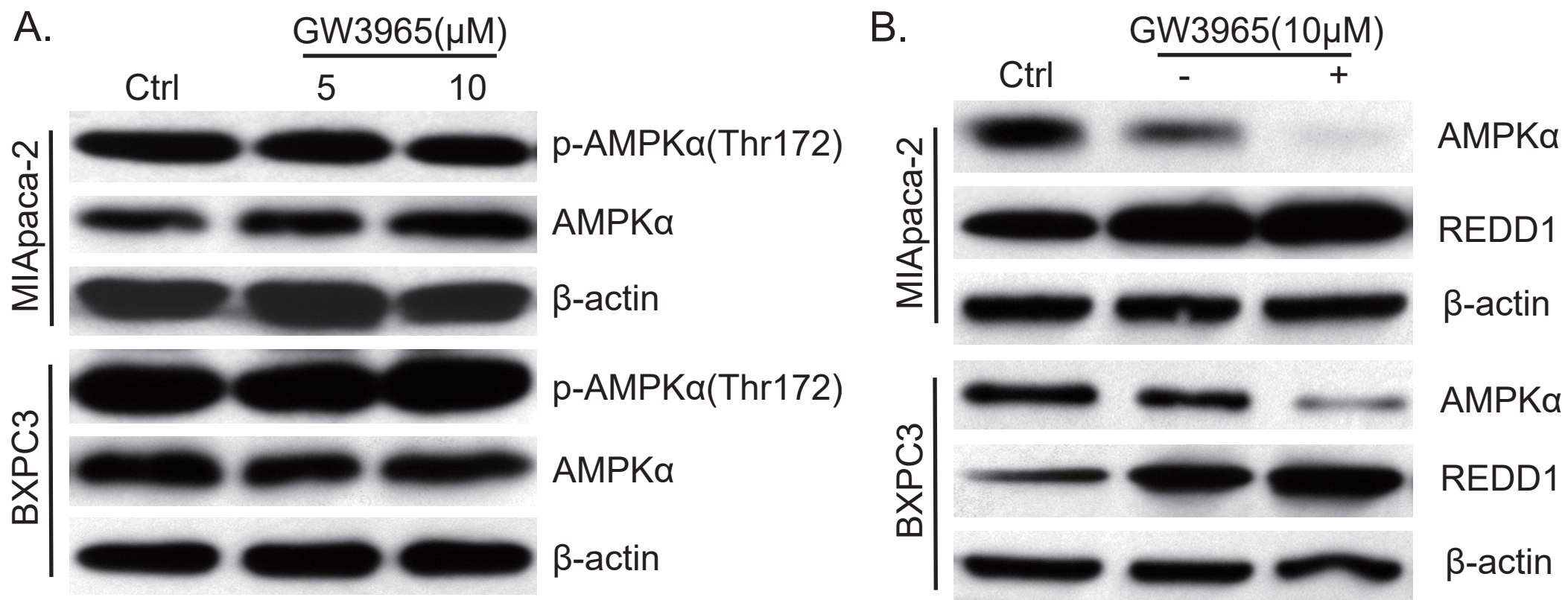

Supplement: Supplementary file 3 — Supporting information. [file CAI2-1-55-s003.pdf]
