# Supplementary material for: ATF4/TXNIP/REDD1/mTOR signaling mediates the antitumor activities of liver X receptor in pancreatic cancers
Source: Cancer Innov. 2022 Jun 30;1(1):55–69. doi: 10.1002/cai2.12 (PMC10686145; doi:10.1002/cai2.12)
Supplement: Supplementary file 4 — Supporting information. [file CAI2-1-55-s001.pptx]

## Slide 1
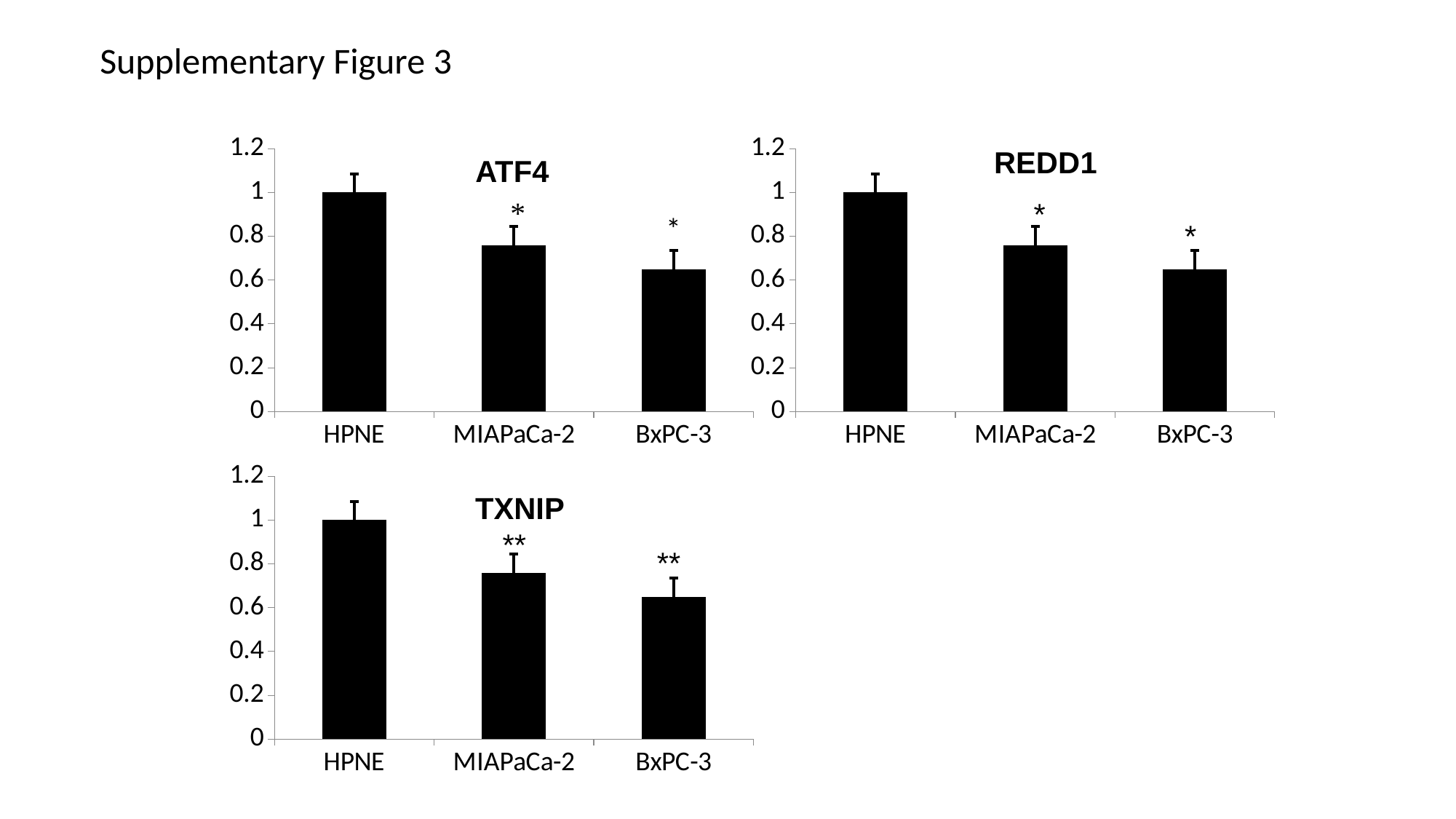

Supplementary Figure 3
### Chart
| Category | |
|---|---|
| HPNE | 1.0 |
| MIAPaCa-2 | 0.76 |
| BxPC-3 | 0.65 |
### Chart
| Category | |
|---|---|
| HPNE | 1.0 |
| MIAPaCa-2 | 0.76 |
| BxPC-3 | 0.65 |REDD1
ATF4
*
*
### Chart
| Category | |
|---|---|
| HPNE | 1.0 |
| MIAPaCa-2 | 0.76 |
| BxPC-3 | 0.65 |TXNIP
**
**
